# A qualitative study exploring patient motivations for screening for lung cancer

**DOI:** 10.1371/journal.pone.0196758

**Published:** 2018-07-05

**Authors:** Joshua A. Roth, Lisa Carter-Harris, Susan Brandzel, Diana S. M. Buist, Karen J. Wernli

**Affiliations:** 1 Hutchinson Institute for Cancer Outcomes Research, Fred Hutchinson Cancer Research Center, Seattle, Washington, United States of America; 2 Comparative Health Outcomes, Policy, and Economics Institute, University of Washington, Seattle, Washington, United States of America; 3 School of Nursing, Indiana University, Indianapolis, Indiana, United States of America; 4 Health Stories Project Insights, Seattle, Washington, United States of America; 5 Kaiser Permanente Washington Health Research Institute, Kaiser Permanente Washington, Seattle, Washington, United States of America; West Virginia University, UNITED STATES

## Abstract

**Background:**

Low-dose computed tomography (LDCT) of the chest for lung cancer screening of heavy smokers was given a ‘B’ rating by the U.S. Preventive Services Task Force (USPSTF) in 2013, and gained widespread insurance coverage in the U.S. in 2015. Lung cancer screening has since had low uptake. However, for those that do choose to screen, little is known about patient motivations for completing screening in real-world practice.

**Objective:**

To explore the motivations for screening-eligible patients to screen for lung cancer.

**Methods:**

Semi-structured qualitative interviews were conducted with 20 LDCT screen-completed men and women who were members of an integrated mixed-model healthcare system in Washington State. From June to September 2015, participants were recruited and individual interviews performed about motivations to screen for lung cancer. Audio-recorded interviews were transcribed and analyzed using inductive content analysis by three investigators.

**Results:**

Four primary themes emerged as motivations for completing LDCT lung cancer screening: 1) trust in the referring clinician; 2) early-detection benefit; 3) low or limited harm perception; and 4) friends or family with advanced cancer.

**Conclusion:**

Participants in our study were primarily motivated to screen for lung cancer based on perceived benefit of early-detection, absence of safety concerns, and personal relationships. Our findings provide new insights about patient motivations to screen, and can potentially be used to improve lung cancer screening uptake and shared decision-making processes.

## Introduction

Approximately 220,000 Americans are diagnosed with lung cancer annually, and only 16.8% survive 5 years following diagnosis.[1] This poor survival prognosis is primarily attributable to the fact that the majority of lung cancer cases have historically been diagnosed at an advanced/distant stage due to the asymptomatic nature of the disease at localized stages[2]. Annual screening with low-dose computed tomography (LDCT) has emerged as a strategy to detect lung cancer earlier and improve survival prognosis—largely based on the significant improvements in stage distribution at diagnosis and lung cancer specific mortality demonstrated by the National Lung Screening Trial (NLST)[3].

In December 2013, based on evidence from a systematic review of lung cancer screening and simulation modeling studies largely informed by findings form the NLST, the U.S. Preventive Services Task Force (USPSTF) recommended lung cancer screening for asymptomatic men and women, aged 55–80 years, with ≥30 pack-year history of smoking, who are current smokers or quit within the last 15 years (grade “B” recommendation)[4]. This recommendation is important because the Affordable Care Act mandates coverage of preventive services with Grade “A” or “B” recommendations from the USPSTF without any patient cost sharing (i.e. co-pays or co-insurance). Additionally, the Centers for Medicare and Medicaid Services (CMS) issued a national coverage determination for LDCT lung cancer screening in February of 2015. As a result of these changes in insurance coverage policy, an estimated 8–9 million Americans gained coverage for LDCT lung cancer screening in 2015[5]. Nonetheless, implementation of screening programs has been slow, with a small number of programs established in or before 2015[6], and many programs still being established.

As healthcare systems across the U.S. develop and implement lung cancer screening programs, it is critical that they carefully consider how to educate patients about screening and engage eligible patients in shared decision-making about the imaging test. In a prior study, we examined the reasons screening-eligible individuals decided to opt out of screening after receiving a recommendation from their primary care provider in a large integrated healthcare system [7]. In this study, we build on our earlier research by exploring patient motivations for agreeing to receive screening for lung cancer in the same healthcare system. The study’s findings can provide valuable insights into the implementation of lung cancer screening from the patient perspective to inform strategies to improve lagging screening uptake[8] and development of patient-centered screening processes.

## Materials and methods

### Study design overview & setting

We conducted a qualitative study to explore patient motivations for pursuing lung cancer screening among members of Kaiser Permanente Washington—a mixed model healthcare system based in Seattle, Washington that both insures and provides medical care to approximately 660,000 members in Washington State. In January 2015, Kaiser Permanente Washington implemented a population-based lung cancer screening program that was designed to identify screening-eligible participants through primary care encounters. In support of the launch, healthcare teams updated smoking history (i.e., pack-year smoking) to identify potentially eligible men and women for clinical care, tools for shared decision were implemented, and an electronic health record (EHR) registry was created to document clinical encounters with potentially screening-eligible patients. Kaiser Permanente Washington had provided screening LDCT scans of the chest to approximately 1,000 members at the time that participants were recruited for this study.

### Recruitment of participants

Eligible participants were randomly selected from members who met USPSTF screening criteria (i.e. age 55–77, ≥30 smoking pack years, and current smoker or quit in the past 15 years) and received LDCT screening within past 60 days (at the time of recruitment). All eligible adults had a screening assessment as 1 (‘negative’) or 2 (‘benign appearance or behavior’) from the American College of Radiology Lung Imaging Reporting and Data System (‘Lung-RADS^™^”). Eligibility was restricted to those with ‘negative’ or ‘benign appearance or behavior’, because these individuals represent the most common lung cancer screening outcome (e.g. 89.4% of results with Lung-RADS[9]), and the study investigators wanted to avoid causing unnecessary stress or concern in patients awaiting diagnostic follow-up related to a positive screening test.

We used EHR to identify 25 potential participants meeting study eligibility criteria. Potential participants were mailed invitation letters describing the study, including a $2 pre-incentive, and noting that a trained interviewer would soon call to ask if they wished to participate. The letter included a telephone number to proactively opt out of study participation, but none opted out. Among the 25 potential participants contacted, we enrolled and interviewed 20 participants (5 declined participation), yielding an 80% participation rate. Each participant who completed the interview was provided with a $20 post-interview cash incentive.

### Data collection

We developed a semi-structured interview guide (Table 1 and S1 File) based largely upon interview questions used in prior qualitative studies focused on cancer screening[10, 11], and refined through an iterative design process involving all study investigators and the Kaiser Permanente Washington survey department. Trained interviewers conducted in-depth semi-structured telephone interviews to elicit participant perspectives on the motivators for lung cancer screening. Participants were also administered several structured multiple choice survey questions to understand their perspectives on the risk of developing lung cancer relative to individuals of a similar age that never smoked and the risk of false-positive results occurring from the screening LDCT scan. The telephone interviews were digitally recorded and transcribed verbatim by a secure, professional transcription service.

**Table 1:** Sample items from the semi-structured interview guide. The full semi-structured interview guide is linked in S1 File.

What made you to think about getting lung cancer screening?
How did you learn that lung cancer screening is offered at Group Health?
Can you tell me about the conversation when you first discussed lung cancer screening with your medical provider?
Who encouraged you to have the screening? Anyone else?
Before the screening, what did you think were the potential benefits for you of receiving lung cancer screening?
Before the screening, what did you think were the potential harms for you of receiving lung cancer screening?
Which potential benefits and harms most affected your decision to receive lung cancer screening?

### Analysis

Three study investigators (JR, KW, SB) analyzed the interview transcripts using standard content analytic methods[12]. The research team—including experts in lung cancer screening behavior, cancer epidemiology, and health services research—iteratively reviewed transcripts and developed a shared coding schema that could be applied with a high level of inter-rater reliability. After independently coding all transcripts, researchers met to discuss themes that emerged and to evaluate the degree of congruence between coding, themes, and classifications. Discrepancies were discussed until consensus was reached. Using this process, we reached saturation of the interview data by the time we completed 20 interviews.

Summary statistics (means and standard deviations) were calculated to analyze the structured survey questions about lung cancer and false-positive result risk.

### Ethical considerations

This study was approved by the Kaiser Permanente Washington Human Subjects Research Committee prior to participant recruitment. Participants provided verbal consent before being interviewed.

## Results

### Participants

Among the 20 participants, median age was 68.0 years (interquartile range = 65.3, 70.3), 40% were male, 90% were Caucasian, and 35% were current smokers (Table 2 and S1 Table).

**Table 2:** Study participant socio-demographic characteristics and smoking history. Patient-level characteristics are linked in S1 Table.

Variable	Count	Proportion
**Gender**		
Male	8	40%
Female	12	60%
**Age**		
55–64	5	25%
65–74	13	65%
≥75	2	10%
**Race**		
White	18	90%
Other Race	2	10%
**Smoking Status**		
Current Smoker	7	35%
Former Smoker	13	65%
**Smoking Pack Years**		
30–39	6	30%
40–49	10	50%
≥50	4	20%

### Motivations for completing lung cancer screening

Four main themes were identified as motivations to complete lung cancer screening: 1) trust in the referring clinician, 2) benefit of early-detection of lung cancer, 3) low or limited harm from LDCT scan perception, and 4) experiences of friends or family with advanced cancer. Each theme is described in detail below and representative quotes for each theme are presented in Table 3 (all representative quotes are provided in S2 Table).

**Table 3:** Major study themes and example quotes. All quotes supporting each theme are linked in S2 Table.

Theme #	Theme Description	Example Quotes
**1**	Trust in the Referring Physician	*Quote 1A*: *“I like my doctor immensely and trusted her*, *and she gave me the wide berth of discussing it (screening) and then when I came back for the next visit…we discussed it some more*. *She’s an awesome doctor that trusts me*, *but also encourages me to do the right thing*. *I think it’s a lot in having trust in your doctor and the way that they broach it*.*”*
		*Quote 1B*: *“They should never belittle you or put you down*, *because that doesn’t help you accomplish what you need to do*. *And nagging doesn’t help you either*, *but they need to talk to you and show real concern and let you know—like my doctor did honestly with me—about my odds*.*”*
**2**	Early-Detection Benefit	*Quote 2A*: *“Even though I am very active and have great health—vitals and everything—I have a concern and actually a fear that I will get lung cancer…it (screening) would detect it (lung cancer) if I had it*, *and perhaps save my life by catching it early”*
		*Quote 2B*: *“Early diagnosis of any significant lung disease would be a huge benefit*. *The earlier you find it*, *the better your chances of treating it*.*”*
**3**	Low or Limited Harm Perception	*Quote 3A*: *“Harms*? *I don’t see any harm in it (screening)*. *The harm would come in not doing the screening*.*”*
		*Quote 3B*: *“I’ve heard about overexposure to radiation and I didn’t want to do that*. *And then…the doctor told me about the low-dose aspect of this and it was a very minimal exposure*, *so that kind of made up my mind that it was worth giving it a shot*.*”*
**4**	Friends or Family with Advanced Cancer	*Quote 4A*: *“I told her (doctor) I wasn’t going to go through what my mother did*. *My mother had lung cancer*. *They gave her chemo and radiation*. *I think it’s a terrible*, *terrible sickness*.*”*
		*Quote 4B*: *“My father died of lung cancer*, *and my husband died of lung cancer*. *So when the doctor suggested this (screening)*, *he didn’t have to do very much with me—I said yes I’ll do it*.*”*

#### Theme 1: Trust in the referring clinician

Most participants (n = 17, 85%) indicated that they were not aware of the availability of lung cancer screening prior to discussion with their clinician. Therefore, referring clinicians’ discussion in a shared decision making consultation encouraged participants to consider screening for lung cancer. Several participants described how their high degree of trust in their clinician motivated them to schedule and complete a screening LDCT scan (Table 3, Quote 1A), and mentioned specific communication styles that were effective in making a referral for a screening LDCT (Table 3, Quote 1B).

#### Theme 2: Early-detection benefit

The majority of participants (n = 15, 75%) described detection of early stage lung cancer as the major motivator for completing lung cancer screening. As demonstrated in interview excerpts (Table 3), participants described screening as a means to detect lung cancer at a localized stage (i.e., to find cancer early) when there are more treatment options and survival prognosis is generally more favorable versus advanced stage. Given that nearly all (n = 18, 90%) reported no knowledge of lung cancer screening prior to the shared decision-making discussion with their provider, this information was likely transferred through communication during the clinical encounter and printed materials shared during the visit. In a structured survey item about perception of personal lung cancer risk, most participants (n = 18, 90%) indicated that they believed themselves to be at ‘higher’ risk of developing lung cancer compared to others their age that never smoked—perhaps explaining why so many participants raised the issue of early-detection and indicated that it was an important motivation for pursuing screening. Among the remaining 2 participants, 1 rated their lung cancer risk as the ‘same’ compared to others their age who never smoked, and 1 abstained from answering the question.

#### Theme 3: Perception of low or limited harm of LDCT exam

Less than half of participants (n = 9, 45%) discussed any potential harm of LDCT exam screening (e.g. Table 3, Quote 3A). Among harms mentioned, participants primarily discussed radiation exposure (n = 8, 40%, e.g. Table 3, Quote 3B). Interestingly, among those who mentioned the issue of radiation exposure, 63% (5 of 8) noted that lower radiation dose involved in LDCT versus other screening modalities (e.g. standard chest CT or PET) motivated them to pursue screening (Table 3, Quote 3B). Only two participants (10%) mentioned the harm of a high false-positive rate with LDCT, which can result in unnecessary invasive diagnostic follow-up procedures and/or anxiety about having lung cancer in the months before diagnostic follow-up procedures. Accordingly, when asked to discuss the benefit-risk tradeoffs of LDCT screening, all participants (n = 20, 100%) expressed the belief that screening benefits outweighed harms (Table 3).

#### Theme 4: Friends or family with advanced cancer

Several participants (n = 6, 30%) described family members’ and friends’ experiences with advanced cancer as a motivation to care for their own health and participate in lung cancer screening. (Table 3, Quotes 4A and 4B). Their motivating stories often focused on family members who died of cancer (especially lung cancer), participants’ perceptions of their loved one’s low quality of life at the end of life due to cancer, and their desire to avoid a similar fate.

## Discussion

Understanding patient motivations for completing lung cancer screening is essential because it provides insight on factors that are most important to screening-eligible patients who follow through with a screening recommendation. This information, combined with an understanding of why individuals opt out of screening[7], is critical as interventions are developed to support patients in the decision-making process in lung cancer screening. Key components of the cancer care continuum involve the multilevel influences that result in cancer-related health outcomes[13]. Early detection through screening is a critical component of the cancer care continuum and success of cancer screening in general. Understanding patient motivations to screen for lung cancer can inform both how new screening programs are designed and implemented, and improve processes in existing programs. Our findings indicate that trust in clinician, benefits of early detection, perceived limited harm of the LDCT, and a family and friend’s experiences with cancer were most salient in motivations to screen. Furthermore, at the medical practice level of the framework, the clinical team, clinical encounter, and patient characteristics are critical factors that can impact the process of care—specifically transitions from risk assessment to screening[13]. Our findings from interviews with Kaiser Permanente Washington members who recently completed screening are theoretically consistent with this view of the cancer care continuum and were grouped into the following four primary themes: 1) trust in the referring clinician; 2) early-detection benefit; 3) low or limited harm perception; and 4) friends or family with advanced cancer as major motivators for completing screening. Our findings extend the work of others[10, 11, 14–16] by explicitly focusing on motivations for completing lung cancer screening from the perspective of the patient, providing new insights into screening behavior that can be used to develop patient-centered lung cancer screening outreach efforts and processes. Patient-centered processes have the potential to improve the continuum of cancer care as it relates to lung cancer. In addition, the results of this study reinforce the importance of these themes in motivating cancer screening in general as our findings are consistent with motivations to screen for other types of cancer such as breast and colorectal[17, 18].

To our knowledge, this is the first study to explicitly explore patient motivations for pursuing lung cancer screening in a community setting. Prior studies have evaluated reasons for opting out of lung cancer screening[7], perceptions of screening processes in participants in the NLST[19], and smoking cessation motivations and perceptions in members of the Veterans Health Administration lung cancer screening demonstration project[11]. Though these prior studies were focused on different aspects of lung cancer screening and/or sub-groups, many similar themes were noted. For example, Carter-Harris et al. also found that all heavy smokers that were interviewed for a qualitative evaluation of reasons for opting out of lung cancer screening initially learned about lung cancer screening from their clinician[7]. Furthermore, Park et al. found that NLST participants also referenced the experiences of their family and friends with lung cancer when reflecting on their risk of developing lung cancer[10]. Zeliadt et al. found that ‘screening provides an external agent in contrast to internal locus of control’—a concept echoed in our finding that many participants were motived to screen by the recommendation of their clinician[11]. These similar qualitative findings suggest that the motivations documented in this study may be common in alternative screening populations too. Additionally, in a quantitative analysis of the National Cancer Institute’s Health Information National Trends Survey, Carter-Harris et al. found that a family history of cancer was associated with increased odds of having discussions about lung cancer screening with a primary care provider (Odds Ratio = 2.53, 95% Confidence Interval = 1.43–4.48)[20]. This result is consistent with our finding that a family history of cancer was an important motivation to complete lung cancer screening.

This study has several important implications for improving the shared decision-making process in lung cancer screening. First, the themes identified can be used to improve patient-provider communication. One required component for lung cancer screening to be reimbursed by Medicare is the shared decision making and counseling visit. In our study, we found participants seemed to readily understand the concept of screening early-detection benefit, and they discussed it as the most prominent theme that motivated screening. This finding suggests that patient-provider discussions about lung cancer screening might benefit from focus on the potential impacts of early-detection of lung cancer, such as ability to receive curative intent treatment, potential to avoid chemotherapy, improved survival prognosis, and improved health-related quality of life. Still, such efforts should carefully balance the discussion of screening benefits with appropriate consideration of screening harms (e.g. false-positives, overdiagnosis, and radiation exposure), as our findings suggest that many screening participants may not understand or retain such information.

Second, we found that many study participants discussed their trust in their clinician as a major motivator for pursuing screening. Participants described open and honest communication with their physician, highlighting the importance of the patient-physician relationship in the decision to complete lung cancer screening. Furthermore, several study participants who were current smokers noted that the relationship with their physician was benefitted by thoughtful discussion of smoking and the potential benefits of cessation, rather than belittling them. Considered in combination with our previous finding that smoking-related stigma in clinical care can serve as a barrier to lung cancer screening[7], this finding suggests the patient-provider relationship can be a primary driver of health behavior (i.e., positively or negatively). Screening-eligible patients with long-standing relationships with their physicians in which patients note a trustworthy relationship, may be more likely to complete screening when recommended. Additionally, patients who have not established a trusting relationship with their physician may discount lung cancer screening recommendations more[21]. We acknowledge that there are many competing issues for patient-physician discussion—particularly in a primary care setting with long-term heavy smokers. Nonetheless, this is one area where targeted effort to establish open rapport may result in large long-term dividends in terms of patient-level health gains.

Clinicians need tools and strategies to systematically communicate the benefits and harms of screening in a consistent manner—such as the University of Michigan ‘Should I Get Screened’ tool[22], AHRQ ‘Is Lung Cancer Screening Right for Me?’ tool[23], and Memorial Sloan Kettering ‘Lung Cancer Screening Decision Tool’[24]. Additionally, connecting the discussion of the importance of screening to the patient’s personal experience of family and friends with advanced cancer might make the importance of screening and smoking cessation more salient for patients and open the door to consideration among those who may be otherwise resistant.

As with all studies, the results should be interpreted within the context of its limitations. First, this was a cross-sectional study evaluating motivations to screen for lung cancer. Therefore, study participants had only completed the initial screening LDCT exam. It is unclear if motivations for initiating participation in a lung cancer screening program will influence both follow-up as well as adherence to annual screening. Future longitudinal research is needed to examine adherence rates among patients who start a lung screening program and explore motivations for both adherence and non-adherence from the patient perspective. Second, this study was conducted in a single mixed-model healthcare system in the Pacific Northwest and was restricted to members who spoke English. As such, participants were insured, predominately Caucasian, and all spoke English fluently. Future studies should evaluate if motivators for completing lung cancer screening are similar in underinsured population, other race/ethnic groups, and among patients with language barriers. Lastly, the majority of the interviews in this study took place 30 to 60 days after screening negative LDCT exam. As a result, recall of some motivations for completing screening may have been limited.

## Conclusion

The participants in this study were motivated to obtain lung cancer screening based on perceived benefit of early-detection, an absence of safety concerns, personal relationships. Our findings provide new insights about patient motivations for pursuing lung cancer screening, and can be used to improve shared decision-making processes among patients and providers engaging in discussions about lung cancer screening.

## Supporting information

S1 File This is the full semi-structured interview script. (DOC)

S1 Table This is the complete list of study participant demographic and smoking status variables and structured interview question responses. (DOCX)

S2 Table This is the complete list of study themes and representative quotes. Each quote within each theme is from a different study participant.
(DOCX)
