# Supplementary material for: A qualitative study exploring patient motivations for screening for lung cancer
Source: PLoS One. 2018 Jul 5;13(7):e0196758. doi: 10.1371/journal.pone.0196758 (PMC6033377; doi:10.1371/journal.pone.0196758)
Supplement: S1 File — (DOC) [file pone.0196758.s001.doc]

**Introduction**

Thank you for being willing to share your opinions and experiences about your lung cancer screening with me. Before we start, I want to assure you that your interview answers will be kept confidential and will not be shared with your health care team. Your feedback will be combined with that of other study participants and will not be linked to your name. You may skip any questions you don’t care to answer and you may end the interview at any time.

We would like to record this interview to make sure we listed your answers correctly. We will share the recording with a trusted company for transcription. They will write down what you said and send the files back to us. Your name will not be associated with the recording or what has been transcribed; a study ID # will be assigned instead. We will destroy the recording when we are finished with the study. In order to help protect your privacy, please do not to state your name while the recorder is on. If your name or other information that identifies you does come up in the written transcripts, we will delete it before sharing with the study researchers.

Do I have your permission to begin the recording for this call?

**[If NO, end call and thank participant for their time] [If YES, continue with interview]**

Thank you. I’m going to start recording now.

**Now that the recording has started, please say “Yes” to confirm that you approve of me recording this interview.**

The first question is about your health in general.

**Section 1: Opening Question**

1. How would you describe your health? Would you say your health is… **[read choices aloud and instruct participant to choose one]**
   1. Poor
   2. Fair
   3. Good
   4. Very Good
   5. Excellent

Based on Group Health records, I understand that you recently received a lung cancer screening test. This test was a CAT scan, also known as a “low-dose CT”. Now I’m going to talk with you about that screening test.

**Section 2: Reasons for seeking screening**

First, I’d like to ask you about your reasons for getting lung cancer screening:

1. What made you to think about getting lung cancer screening?
   1. How did you learn that lung cancer screening is offered at Group Health?
   2. Can you tell me about the conversation when you first discussed lung cancer screening with your medical provider?
2. Who encouraged you to have the screening?
   1. Anyone else?

**Section 3: Assessing risk perception constructs**

1. Before the screening, what did you think were the potential benefits for you of receiving lung cancer screening?
2. Before the screening, what did you think were the potential harms for you of receiving lung cancer screening?
3. Before the screening, did any of your medical providers discuss the potential benefits or harms of lung cancer screening with you?

- **[IF NO🡪move to next question**]
- **[IF YES]** What specific benefits and harms did your medical providers discuss with you?

1. Which potential benefits and harms most affected your decision to receive lung cancer screening?
2. Suppose there were 100 people who received a screening result showing that they may have lung cancer. How many do you estimate would **actually** have lung cancer?

**Section 4: Impressions of screening**

1. What was it like for you to have lung cancer screening?
2. How did you feel while you were waiting to get the screening results?

**Section 5: Receiving test results**

Now I’d like to ask you about your test results. **[Ask the following questions about the initial screening test, not any follow-up imaging in response to initial test]**

1. How did you find out the result of your lung cancer screening?
2. Did anyone explain the meaning of these results to you? **[IF NO🡪move to next question]**
   1. Probe: How were the results of your lung cancer screening explained to you?
   2. What questions, if any, did you ask when you received the test results?
3. Can you describe how you felt about your health and wellbeing when you got your screening test results?
4. What, if any, lifestyle changes have you made because of your screening result?

- **[IF NO]** Do you think you might make any lifestyle changes in the next year?
- **[IF YES]** Probe: What additional lifestyle changes might you make in the next year?

1. Do you think you will continue to get lung cancer screening if your doctor recommends it?

- **[IF NO]** Probe: Why not?
- **[IF YES]** Probe: Why?

The next section is about smoking and health.

**Section 6: Smoking risks**

1. Suppose there were 100 people who started smoking regularly at age 18, how many do you estimate would develop lung cancer in their lifetime?
2. Compared to other **[use participant’s gender]** men/women your age that never smoked, do you think your risk of developing lung cancer is… **[read choices aloud and instruct participant to choose one]**
   1. Higher
   2. Lower
   3. The Same
3. Compared to other **[use participant’s gender]** men/women your age that never smoked, do you think your risk of having a heart attack is… **[read choices aloud and instruct participant to choose one]**
4. Higher
5. Lower
6. The Same

**Section 7: Final Feedback**

1. Do you have any recommendations for health care providers about how to improve the experience of people getting screened for lung cancer?

Those are all the questions I have for you today. Is there anything else you would like to share with the research team about lung cancer screening or this study?
