# Supplementary material for: A qualitative study exploring patient motivations for screening for lung cancer
Source: PLoS One. 2018 Jul 5;13(7):e0196758. doi: 10.1371/journal.pone.0196758 (PMC6033377; doi:10.1371/journal.pone.0196758)
Supplement: S1 Table — (DOCX) [file pone.0196758.s002.docx]

**Table S1: Complete list of study participant demographic and smoking status variables and structured interview question responses.**

| **Participant #** | **Age at Screening** | **Gender** | **Race** | **Smoking Pack Years** | **Smoking Status at Interview** | **Aware of Lung Cancer Screening** | **Lung Cancer Risk vs. Non-Smokers** | **Screening Benefits Outweigh Harms** |
| --- | --- | --- | --- | --- | --- | --- | --- | --- |
| **1** | 69 | Female | Caucasian | 43 | Quit | No | Higher | Yes |
| **2** | 60 | Female | Caucasian | 35 | Quit | No | Higher | Yes |
| **3** | 70 | Female | Caucasian | 30 | Quit | No | Higher | Yes |
| **4** | 61 | Male | Caucasian | 30 | Quit | No | Higher | Yes |
| **5** | 68 | Male | Caucasian | 40 | Smoker | Yes | Same | Yes |
| **6** | 71 | Female | Black | 40 | Quit | No | Higher | Yes |
| **7** | 68 | Female | Caucasian | 40 | Smoker | No | Missing | Yes |
| **8** | 66 | Male | Caucasian | 40 | Quit | No | Higher | Yes |
| **9** | 76 | Male | Caucasian | 38 | Smoker | No | Higher | Yes |
| **10** | 72 | Female | Black | 50 | Quit | No | Higher | Yes |
| **11** | 59 | Female | Caucasian | 41 | Quit | No | Higher | Yes |
| **12** | 70 | Female | Caucasian | 42 | Quit | Yes | Higher | Yes |
| **13** | 70 | Female | Caucasian | 38 | Quit | No | Higher | Yes |
| **14** | 57 | Male | Caucasian | 30 | Quit | No | Higher | Yes |
| **15** | 68 | Female | Caucasian | 46 | Smoker | No | Higher | Yes |
| **16** | 63 | Female | Caucasian | 62 | Smoker | No | Higher | Yes |
| **17** | 66 | Male | Caucasian | 46 | Smoker | Yes | Higher | Yes |
| **18** | 68 | Male | Caucasian | 75 | Smoker | No | Higher | Yes |
| **19** | 73 | Male | Caucasian | 50 | Quit | No | Higher | Yes |
| **20** | 75 | Female | Caucasian | 45 | Quit | No | Higher | Yes |
