# Supplementary material for: A qualitative study exploring patient motivations for screening for lung cancer
Source: PLoS One. 2018 Jul 5;13(7):e0196758. doi: 10.1371/journal.pone.0196758 (PMC6033377; doi:10.1371/journal.pone.0196758)
Supplement: S2 Table — Each quote within each theme is from a different study participant. (DOCX) [file pone.0196758.s003.docx]

**Table S2: Complete list of study themes and representative quotes. Each quote within each theme is from a different study participant.**

| **Theme #** | **Theme Description** | **Example Quotes** |
| --- | --- | --- |
| 1 | Trust in the Referring Physician  N=8 | *Quote 1A: “I like my doctor immensely and trusted her, and she gave me the wide berth of discussing it (screening) and then when I came back for the next visit…we discussed it some more. She’s an awesome doctor that trusts me, but also encourages me to do the right thing. I think it’s a lot in having trust in your doctor and the way that they broach it.”*  *Quote 1B: “They should never belittle you or put you down, because that doesn’t help you accomplish what you need to do. And nagging doesn’t help you either, but they need to talk to you and show real concern and let you know—like my doctor did honestly with me—about my odds.”*  *Quote 1C: I’ve been with the same doctor for quite a while and so if she wants me to do something, I’ll just go ahead and do it.”*  *Quote 1D: “I smoked for 45 years, so when the doctor suggested this, he didn’t have to do very much with me, I said yes.”*  *Quote 1E: “If she (the doctor) recommends it (screening), I definitely will do it. If she recommends it, it’s something she thinks is important”.*  *Quote 1F: “I’ve heard of overexposure to radiation and I didn’t want to do that. Then he (the doctor) told me…about the low-dose aspect of this and that it is minimal exposure, so that kind of made up my mind that it was worth giving it a shot.”*  *Quote 1G:* *“I trust my doctor so much for one thing…So if my doctor suggested I have another one (screening), I would definitely do that.”*  *Quote 1H: “I had no objection to it (screening). I was aware that I was probably overdue for a screening. He (the doctor) recommended it and I readily agreed.”* |
| 2 | Early-Detection Benefit  N=15 | *Quote 2A: “Even though I am very active and have great health—vitals and everything—I have a concern and actually a fear that I will get lung cancer…it (screening) would detect it (lung cancer) if I had it, and perhaps save my life by catching it early”*  *Quote 2B: “Early diagnosis of any significant lung disease would be a huge benefit. The earlier you find it, the better your chances of treating it.”*  *Quote 2C: “There was the potential benefit of discovering it early, while you could still do something”*  *Quote 2D: “I want to know what happened to that spot they found on my lung.”*  *Quote 2E: “I had smoked for 45 years, so there does exist a possibility that I may end up with lung cancer. The earlier it’s detected, the better it would be for my chances of survival.”*  *Quote 2F: “Your odds of getting cancer because you smoke is pretty great and I’ve been smoking since I was 16 years old and I’m 63...When she mentioned doing the CT scan, to see if I might have cancer, that’s when I said ok.”*  *Quote 2G: “[Screening can] give us some information as to what your (cancer) status is at the time”*  *Quote 2H: “The benefit (of screening) was early detection... Cancers run in our family and if I can do something to gain an edge on that, then I will do what I can do.”*  *Quote 2I: “If they found it (cancer) early enough, they might be able to fix it without me dying.”*  *Quote 2J: “The benefit of finding a cancerous growth and after all my years of heavy smoking…the chance to find it early was just so beneficial to me.”*  *Quote 2K: “I realize catching things (cancer) early is much preferable to waiting and finding out later.”*  *Quote 2L: “That it (screening) would detect if I had it (cancer) and perhaps save my life by catching it early.”*  *Quote 2M: “Well, obviously early diagnosis of any significant lung disease would be a huge benefit. The earlier you find it, the better your chances of treating it.”*  *Quote 2N: “I just think it’s a great tool that they have added and I really think it (screening) will help either identify lung cancer in advance, or at least in time for them to actually do something before it becomes too late.”*  *Quote 2O:* *“If something (cancer) does show up (in screening), you catch it early enough to follow through (with treatment).”* |
| 3 | Low or Limited Harm Perception  N=9 | *Quote 3A: “Harms? I don’t see any harm in it (screening). The harm would come in not doing the screening.”*  *Quote 3B: “I’ve heard about overexposure to radiation and I didn’t want to do that. And then…the doctor told me about the low-dose aspect of this and it was a very minimal exposure, so that kind of made up my mind that it was worth giving it a shot.”*  *Quote 3C: “I didn’t think there would be any harm”*  *Quote 3D: “I don’t think there is any harm in it (screening)”*  *Quote 3E: “I know there’s always some harm with radiation, but any slight downside is certainty made up for by the upside of knowing what’s going on with my lungs.”*  *Quote 3G:* *“The CAT scan approach (to screening) seems to be very non-invasive, low dose, low risk, so I didn’t see any risks at all in this (screening).”*  *Quote 3H:* *“Nothing (harms) as far as the screening itself. No issues, no fears.”*  *Quote 3K: “No, there’s no harm (in screening).”*  *Quote 3L: “I did not consider any harm.”* |
| 4 | Friends or Family with Advanced Cancer  N=6 | *Quote 4A: “I told her (doctor) I wasn’t going to go through what my mother did. My mother had lung cancer. They gave her chemo and radiation. I think it’s a terrible, terrible sickness.”*  *Quote 4B: “My father died of lung cancer, and my husband died of lung cancer. So, when the doctor suggested this (screening), he didn’t have to do very much with me—I said yes I’ll do it.”*  *Quote 4C: “My mom had lung cancer, which she died of. So did my dad.”*  *Quote 4D: “My father had lymphoma cancer, not lung cancer, but he smoked for a long time also. Cancers run in our family and if I can do something to kind of gain an edge on that, then I want to do what I can do.”*  *Quote 4E: “My sister had lung cancer and died from it quite a few years ago.”*  *Quote 4F:* *“My mom passed away (of cancer) at an early age of 42.”* |
